# Case Report: Successful liver transplantation after achieving complete clinical remission of advanced HCC with Atezolizumab plus Bevacizumab combination therapy

**DOI:** 10.3389/fimmu.2023.1205997

**Published:** 2023-06-12

**Authors:** Yasmina Chouik, Domitille Erard, Hassan Demian, Thomas Schulz, Tessa Mazard, Kerstin Hartig-Lavie, Teresa Antonini, Jean-Yves Mabrut, Kayvan Mohkam, Agnès Rode, Philippe Merle

**Affiliations:** ^1^ Cancer Research Center of Lyon (CRCL), INSERM U1052, Centre National de la Recherche Scientifique UMR5286, Lyon, France; ^2^ Department of Hepatology, Hôpital Croix-Rousse, Hospices Civils de Lyon, Lyon, France; ^3^ Department of General Surgery and Liver Transplantation, Hôpital Croix-Rousse, Hospices Civils de Lyon, Lyon, France; ^4^ Department of Intensive Care, Hôpital Croix-Rousse, Hospices Civils de Lyon, Lyon, France; ^5^ Department of Radiology, Hôpital Croix-Rousse, Hospices Civils de Lyon, Lyon, France

**Keywords:** immunotherapy, case-report, liver transplantation, Atezolizumab, complete response, Bevacizumab, hepatocellular carcinoma

## Abstract

**Background:**

Atezolizumab plus Bevacizumab combination therapy has recently emerged as the new standard of care for unresectable HCC. Significant tumor burden reduction can be observed under that treatment, raising the question of liver transplantation (LT). The safety of another immune checkpoint inhibitor (ICI), nivolumab, is unclear in the pre-transplant setting.

**Method:**

We report the case of a 57-y old man, with initial unresectable multinodular HCC contraindicated to LT and locoregional therapies, who achieves complete tumor response after Atezolizumab/Bevacizumab, and subsequently underwent LT for liver failure.

**Results:**

Explant analysis revealed complete pathological response with no tumor remnant. The patient suffered from several post-operative complications but no HCC recurrence or biopsy-proven acute rejection occurred 10 months after LT.

**Conclusions:**

Atezolizumab/Bevacizumab therapy may enable complete pathological response of advanced HCC. Safety of prolonged treatment need to be assessed.

## Introduction

LT is the optimal treatment for patients with early-stage HCC since it enables to remove both the tumor and the underlying chronic hepatopathy, and gives the highest long-term survival. Due to organ shortage and risk of HCC recurrence after LT, only highly selected patients with low tumor burden are eligible to LT. In patients with HCC beyond the criteria, locoregional therapies, such as TACE or percutaneous ablations, have been validated as effective and safe downstaging strategies to LT (1, 2). The combination of PD-L1 (Atezolizumab) and vascular endothelial growth factors (Bevazicumab) inhibitors has recently emerged as the standard of care for patients with unresectable HCC (3). In this population, this immune-oncology (IO)-based combination improves the median overall survival, with one third of patients having objective response, and 8 to 12% per RECIST v1.1 or mRECIST respectively (3, 4). The efficacy and safety of Atezolizumab plus Bevacizumab combination as a downstaging or bridging therapy to LT remains unknown. Case-report studies have raised red flags about the use of nivolumab, another ICI, in the pre-transplant setting, due to increased risk of acute rejection (5–8). Yet, LT could be a therapeutic option in patients with objective response fulfilling the HCC LT criteria, and/or developing liver failure.

## Case presentation

**Figure 1:**
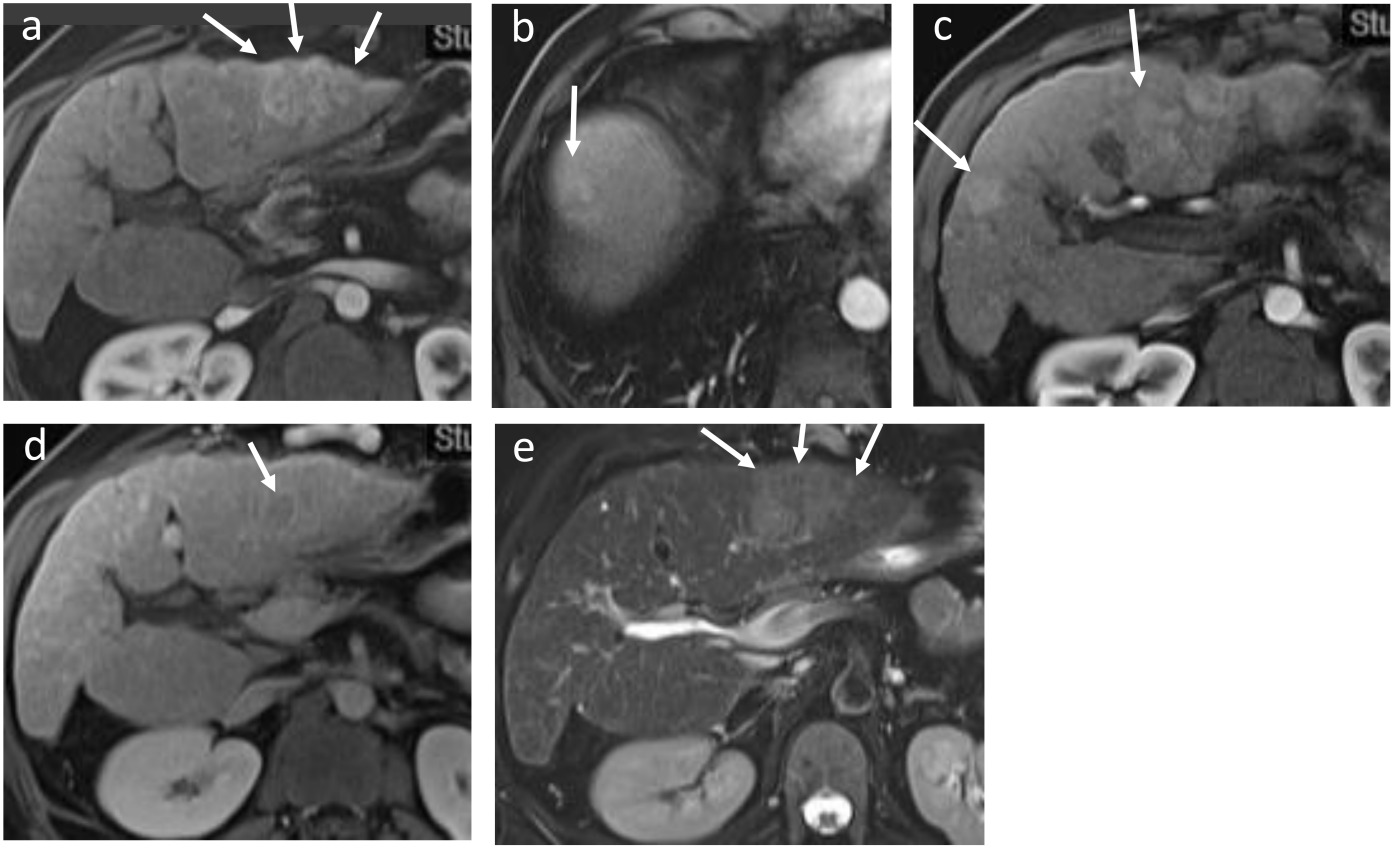
Initial MRI showing multiple hypervascular lesions and no macrovascular tumoral invasion: a main lesion (6 cm in diameter) with late washout on portal phase and slightly hyperintense T2 in the left lobe, and multiple smaller lesions in the right lobe. **(A)** Sequence T1 with injection (arterial phase): main lesion in the left lobe; **(B)** Sequence T1 with injection (arterial phase): multiple hypervascular lesions in the hepatic dome and **(C)** in the segment IV and V. **(D)** Sequence T1 with injection (portal phase): late washout of the main lesion. **(E)** T2 sequence: main left lesion slightly hyperintense T2.

A 57-year-old male patient with alcohol-related cirrhosis was referred to our center in January 2021 for a multinodular HCC BCLC-B. Radiological evaluation by CT and MRI concluded to several bilobar HCC nodules with the largest tumor diameter of 6 cm (**Figure 1**). Initial AFP level was at 379 ng/mL and Child-Pugh score was A6. *Owed to the diffuse and infiltrative nature of the lesions, systemic treatment was validated after discussion in a multidisciplinary tumor board according to updated BCLC strategy* (9). He started Atezolizumab (1200mg Q3W) plus Bevacizumab (15mg/kg Q3W) intravenously in February 2021 and underwent 18 cycles. AFP level normalized after the 5^th^ cycle (5 ng/mL). Follow-up CTs showed progressive decrease in size and devascularization of the nodules. Complete tumoral response per mRECIST and partial response per RECIST v1.1 criteria was achieved in August 2021 after 9 cycles.

**Figure 2:**
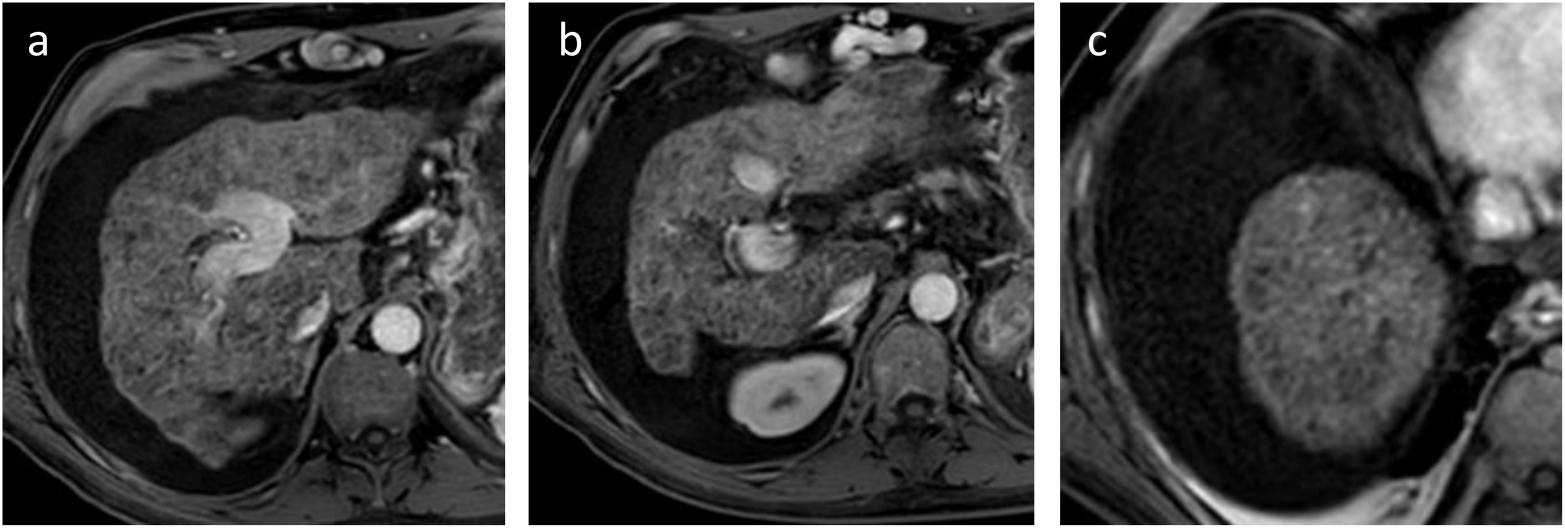
Last follow-up MRI before LT showing complete disappearance of the hepatic lesions, associated with ascitic decompensation (sequence T1 with injection). **(A)** Main lesion in the left lobe; **(B)** segment IV and **(C)** hepatic dome.

From January 2022, liver functions progressively and rapidly worsened. The patient required hospital admission in February 2022, one week after the last IO therapy cycle, for ascites onset. MELD score was 22. No trigger was identified. Liver MRI confirmed complete tumor disappearance (**Figure 2**). Pre-LT choline and FDG-PET scans showed no intra- or extra-hepatic abnormal hypermetabolism, and the patient was listed for LT.

In April 2022, he was admitted in intensive care unit for hepatic encephalopathy. MELD score was 36. After probabilistic antibiotic and antifungal therapy, he was successfully transplanted a few days later. Explant pathology revealed complete tumor necrosis in the two persistent avascular nodules identified per RECIST v1.1, with no tumor remnant. No immune cell infiltration was observed.

Initial post-LT immunosuppression consisted in anti-IL2 receptor monoclonal antibodies, high doses of corticosteroids for 7 days then progressively tapered, tacrolimus at day 1 and mycophenolate mofetil (1g BID). Post-LT course was marked by numerous post-operative complications, including bacterial and fungal infection of peri-hepatic collections, CMV infection, hepatic artery stenosis and anastomotic biliary stricture. *Fifteen* months later, the patient is in good health, with no sign of HCC recurrence. No acute rejection occurred during the early post-transplantation period, with 2 liver biopsies performed at 1 and 3 weeks after LT. The patient is currently under dual immunosuppression and has strictly normal liver tests.

## Discussion

Here, we report the case of a patient with unresectable HCC initially ineligible to curative options, successfully treated by IO-based therapy leading to complete tumor disappearance and allowing subsequent LT for liver failure. Of note, the patient did not show any graft rejection or tumor recurrence to date.

Atezolizumab plus Bevacizumab combination was very recently established as the new standard of care for intermediate HCC not eligible to TACE or advanced HCC with preserved liver functions (3). Complete radiological response rate is estimated at 8% per RECIST v1.1 and 12% per mRECIST (3, 4). The first case of pre-transplant ICI therapy was reported in 2020 (5), describing a fatal severe acute rejection in the early post LT period following nivolumab. Since then, others demonstrated favorable outcome after pre-transplant nivolumab therapy (6, 7). The risk of acute rejection seems to be increased, notably in case of short time between last treatment and LT (6). However, Tabrizian et al. reported 8 cases of LT with patients receiving the last dose of nivolumab within 4 weeks before the surgery, with only one case of mild biopsy-proven acute rejection (BPAR) (7). On the contrary, Qiao et al. found 2 fatal cases of BPAR among 7 patients receiving nivolumab combined with lenvatinib before LT (8). Four weeks has been proposed as the minimum time between the last infusion of nivolumab and LT due to its half-life (7), which is similar to Atezolizumab (10). Here, LT was performed 3 months after the last IO-based therapy, and no BPAR occurred.

In the new therapeutic era of immunotherapy, only two previous reports suggested the interest of Atezolizumab/Bevacizumab as a downstaging therapy to LT (11, 12). Both showed a significant tumor size reduction with persistent tumor lesions, allowing access to LT. To our knowledge, this is the first case demonstrating pathological complete response of HCC after IO-based therapy.

Herein, our patient experienced several early post-operative complications, including infectious events. These complications, expected in this setting of decompensated cirrhosis and poor general status, could nonetheless also be favored by immunotherapy. Concomitantly to tumor burden reduction, liver functions have deteriorated progressively, resulting in the need for LT. Hepatic decompensation may be more frequent in patients treated with Atezolizumab/Bevacizumab compared to other systemic therapies although no high level of evidence has been shown (13). In our recent experience, this clinical presentation is not uncommon in patients treated by IO but the responsibility between the treatment versus the natural history of cirrhosis is still to be determined.

If the temporal sequence between cessation of ICI therapy and LT remains to be determined, the duration of treatment in a complete responder patient must also be investigated in the future. Indeed, it remains unknown how long should patients be kept under IO after obtaining complete response. To assess the impact of IO and VEGF inhibitors on the non-tumor liver parenchyma and liver functions in patients under long-term Atezolizumab/Bevacizumab therapy is necessary.

Altogether, this report confirms the potential efficacy of downstaging strategy by Atezolizumab plus Bevacizumab in HCCs ineligible for curative therapies, and demonstrates the possibility of pathological complete response under this treatment. Safety of this attitude, overall length of pre-transplant systemic treatment and exact timing between IO therapy cessation and LT must be assessed in further clinical trials.

## Data availability statement

The raw data supporting the conclusions of this article will be made available by the authors, without undue reservation.

## Ethics statement

Ethical approval was not provided for this study on human participants since, according to the French law (Loi Jardé), retrospective studies do not require Institutional Review Board approval. The patients/participants provided their written informed consent to participate in this study. Written informed consent was obtained from the individual(s) for the publication of any potentially identifiable images or data included in this article.

## Author contributions

YC, DE, AR and PM wrote the manuscript. All the authors were involved in medical care of the patient and approved the final version of the manuscript. All authors contributed to the article and approved the submitted version.
